# Genomic Characterization of Probiotic Purple Nonsulfur Bacteria *Cereibacter sphaeroides* Strains S3W10 and SS15: Implications for Enhanced Shrimp Aquaculture

**DOI:** 10.3390/life14121691

**Published:** 2024-12-20

**Authors:** Chollachai Klaysubun, Nattarika Chaichana, Sirikan Suwannasin, Kamonnut Singkhamanan, Thunchanok Yaikhan, Duangporn Kantachote, Rattanaruji Pomwised, Monwadee Wonglapsuwan, Komwit Surachat

**Affiliations:** 1 Department of Biomedical Sciences and Biomedical Engineering, Faculty of Medicine, Prince of Songkla University, Songkhla 90110, Thailand; chollachai951@gmail.com (C.K.); naampueng.np@gmail.com (N.C.); sirikan4036@gmail.com (S.S.); skamonnu@medicine.psu.ac.th (K.S.); p_rair@hotmail.com (T.Y.); 2 Division of Biological Science, Faculty of Science, Prince of Songkla University, Songkhla 90110, Thailand; duangporn.k@psu.ac.th (D.K.); rattanaruji.p@psu.ac.th (R.P.); monwadee.wo@psu.ac.th (M.W.); 3 Translational Medicine Research Center, Faculty of Medicine, Prince of Songkla University, Songkhla 90110, Thailand

**Keywords:** probiotics, *Cereibacter sphaeroides*, purple nonsulfur bacteria, bioinformatics, genomic characterization

## Abstract

*Cereibacter sphaeroides* strains S3W10 and SS15, isolated from shrimp ponds, exhibit potential probiotic benefits for aquaculture. In this study, the genomic features of S3W10 and SS15 were thoroughly characterized to evaluate their probiotic properties and safety for aquaculture use. The genomes of S3W10 and SS15 consist of 130 and 74 contigs, with sizes of 4.6 Mb and 4.4 Mb and GC contents of 69.2%. Average nucleotide identity (ANI), digital DNA-DNA hybridization (dDDH), and phylogenomic analyses confirmed that these strains belong to *C. sphaeroides*. Genome annotation predicted 4260 coding sequences (CDS) in S3W10 and 4086 CDS in SS15, including genes associated with stress tolerance, nutrient absorption, and antioxidant activity. Notably, genes related to vitamin B12 synthesis, digestive enzyme production, and carotenoid biosynthesis, which support shrimp health, were identified in both genomes. CAZyme analysis identified 116 and 115 carbohydrate-active enzymes in S3W10 and SS15, respectively, supporting adaptation to gastrointestinal environments and the host immune response. Pan-genome analysis across *C. sphaeroides* strains revealed 7918 gene clusters, highlighting the open pan-genome structure of this species and its high genetic diversity. Further bioinformatic analyses assessing mobile genetic elements, antibiotic-resistance genes, and virulence factors demonstrated the safety of both strains for aquaculture, as no plasmids or virulence genes were identified. The genomic insights in this study provide a deeper understanding of the strains’ adaptability and functional potential, aligning with previous in vitro and in vivo studies and highlighting their potential for use in shrimp cultivation.

## 1. Introduction

*Cereibacter* is a genus within the family Rhodobacteraceae, which are Gram-negative, anoxygenic photosynthetic bacteria found in a wide variety of environments, including aquatic habitats and soil [1]. Currently, the genus *Cereibacter* comprises eight recognized species with validly published names according to the LPSN (List of Prokaryotic names with Standing in Nomenclature) (https://lpsn.dsmz.de/genus/cereibacter, accessed on 1 November 2024). Recent studies support the reclassification of *Rhodobacter sphaeroides* to *Cereibacter sphaeroides* based on genomic evidence, reflecting a more accurate understanding of its phylogenetic relationships and metabolic capabilities [2].

Purple nonsulfur bacterium (PNSB) such as *C. sphaeroides* are known to survive and function in diverse environmental conditions, including the gastrointestinal tracts (GITs) of aquaculture species, due to their metabolic flexibility, stress tolerance, and ability to produce bioactive compounds beneficial to host health. In shrimp aquaculture, the probiotic use of PNSB like *C. sphaeroides* could play a crucial role in disease prevention and growth enhancement. Previous studies have demonstrated that the incorporation of *C. sphaeroides* into shrimp feed has been shown to improve digestive enzyme activities in the GIT of shrimp, thereby facilitating better nutrient absorption and growth performance [3]. Research indicates that the addition of PNSB, including *C. sphaeroides*, to shrimp diets can enhance the shrimp’s tolerance to environmental stressors, such as ammonia and pathogenic *Vibrio* species, ultimately leading to improved survival rates and overall health [4,5]. Furthermore, PNSB can also help in the bioconversion of organic matter, thereby improving water quality and reducing the reliance on chemical inputs, which is crucial given the environmental concerns associated with intensive shrimp farming practices [6].

Genomic analysis has emerged as a powerful tool for understanding the potential of probiotic strains by identifying genes associated with probiotic traits, such as stress resistance, biofilm formation, nutrient synthesis, and antimicrobial activity [7,8]. Whole-genome sequencing also allows for a thorough safety assessment by detecting mobile genetic elements (MGEs), antibiotic-resistance genes (ARGs), and virulence factors that could pose risks if transferred to other organisms [9,10]. This study aimed to determine the draft genomes of two *C. sphaeroides* strains, S3W10 and SS15, obtained from a previous study [3], and to conduct a comprehensive comparative genomic analysis of all bacterial strains within the *C. sphaeroides* species. We performed a pan-genome analysis using a filtered dataset of genomes from the NCBI Database, representing the first meta-analysis of the *C. sphaeroides* species. Additionally, we conducted multiple bioinformatics analyses to investigate genomic features, with a particular focus on identifying genes related to probiotic and safety properties in strains S3W10 and SS15, to assess their potential benefits in shrimp aquaculture.

## 2. Material and Methods

### 2.1. Bacterial Strains

Strains S3W10 and SS15 of *C. sphaeroides* were sourced from a previous study [3]. These strains originated from shrimp pond environments along southern coastlines in Thailand. Bacteria were cultivated in a basic isolation medium (BIM) broth [3] under microaerobic-light conditions for 48 h.

### 2.2. Whole-Genome Sequencing Analysis and Assembly

Genomic DNA from *C. sphaeroides* strains S3W10 and SS15 was extracted and purified using the QIAamp^®^ DNA Mini Kit (QIAGEN, Valencia, CA, USA) in accordance with the manufacturer’s instructions. DNA concentration and quality were measured with a NanoDrop™ 2000/2000c spectrophotometer (Thermo Fisher Scientific, Norristown, PA, USA), while its integrity and purity were confirmed through agarose gel electrophoresis. The purified DNA was prepared for library construction and sequenced on the BGISEQ-500 platform (BGI, Shenzhen, China), generating 150 bp paired-end reads. A total of 1 Gbp of sequence data was obtained. The raw sequencing reads were subsequently *de novo* assembled and annotated using BacSeq tools [11].

### 2.3. Genome Annotation and Visualization

The genomes of *C. sphaeroides* strains S3W10 and SS15 were functionally annotated using rapid annotations using the subsystems technology (RAST) [12] and the EggNOG 5.0 [13] with default settings to identify gene products. The annotated data were examined to detect genes associated with potential probiotic functions, including those supporting bacterial survival in gut environments. A customized graphical representation of the S3W10 and SS15 genomes were created using Proksee (https://proksee.ca, accessed on 1 October 2024) [14]. For assessing mobile genetic elements (MGEs) and prophages, the tools mobileOG-db [15] and Phigaro [16] were used, respectively. Antibiotic-resistance genes (ARGs) were identified by searching against the CARD database [17] and the ResFinder web-based tool (https://genepi.food.dtu.dk/resfinder, accessed on 27 October 2024) [18]. Plasmids were detected using the PlasmidFinder database [19], and virulence factors were screened using the VFDB database [20] with an identity and coverage threshold of 80%. The identification of intact prophages within the genomes was performed using the PHASTER web server [21]. The presence of genomic islands (GIs) and pathogenicity-related genes was assessed using Islandviewer 4 (http://www.pathogenomics.sfu.ca/islandviewer/, accessed on 27 October 2024) [22]. The clustered regularly interspaced short palindromic repeats (CRISPRs) sequences and their associated *cas* genes were identified using CRISPRCasFinder (https://crisprcas.i2bc.paris-saclay.fr/CrisprCasFinder/Index, accessed on 27 October 2024) [23] and CRISPRCasTyper (https://cctyper.crispr.dk/, accessed on 27 October 2024) [24].

### 2.4. Identification of Carbohydrate-Active enZyme (CAZyme)

Genes encoding carbohydrate-active enzymes were identified using dbCAN2 HMMs of CAZy families v10 through the KBase web interface [25,26], applying default parameters.

### 2.5. Detection of Predicted Secondary Metabolite Biosynthetic Gene Clusters (BGCs)

Putative secondary metabolite gene clusters (BGCs) in the genomes of S3W10 and SS15 were predicted using antiSMASH (v 6.0) with default settings (https://antismash.secondarymetabolites.org/, accessed on 1 October 2024) [27]. Additionally, putative bacteriocins were also searched using the BAGEL4 database [28] using blastp with 30% identity and 80% coverage thresholds.

### 2.6. Pan-Genome Analysis

Whole-genome sequences of *C. sphaeroides* available in the NCBI datasets (20 October 2024) were downloaded for analysis. From a total of 35 genomes, 20 were selected after removing those that were unannotated, redundant, or atypical genomes. Genomes with less than 90% completeness or more than 5% contamination were also excluded (Supplementary Table S1). The genome of the purple nonsulphur bacteria *C. sphaeroides* strains S3W10 and SS15 was included among these. The pan-genome analysis was carried out using PanExplorer [29] with GenBank files annotated by Prokka. The workflow involved grouping genes and analyzing the pan-genome with PanACoTA software (version 1.4.1), which handles quality control and builds pan-genomes [30]. In this analysis, a sequence identity threshold of 80% was used for BLAST pairwise alignments, and homolog proteins were clustered using a single-linkage method. A rarefaction curve showing the discovery rate of new gene clusters was generated with the micropan R package (version 4.2.0).

### 2.7. The Average Nucleotide Identity (ANI) and Phylogenetic Analyses

The genetic distance and relatedness of the *C. sphaeroides* genome set, including strains S3W10 and SS15, were assessed through an Average Nucleotide Identity (ANI) analysis using JspeciesWS [31]. Whole-genome phylogenetic analysis was carried out using the Genome BLAST Distance Phylogeny (GBDP) method. This approach generated phylogenetic relationships based on high-resolution whole-genome sequence comparisons. The analysis was performed utilizing the Type Strain Genome Server (TYGS) platform (https://tygs.dsmz.de/, accessed on 27 October 2024) [32].

## 3. Results and Discussion

### 3.1. Genome Characteristics and Annotation of C. sphaeroides S3W10 and SS15 Strains

**Figure 1:**
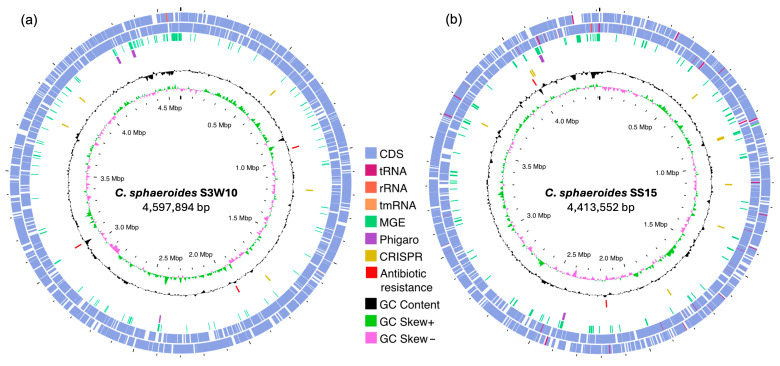
Chromosome maps of *C. sphaeroides* strains S3W10 (**a**) and SS15 (**b**), created using the Proksee v1.0.0a6 server. The maps illustrate the distribution of CDS, tRNA, rRNA, tmRNA, MGEs, phage region predictions, CRISPR-Cas systems, antibiotic-resistance genes, and GC content variation.

**Figure 2:**
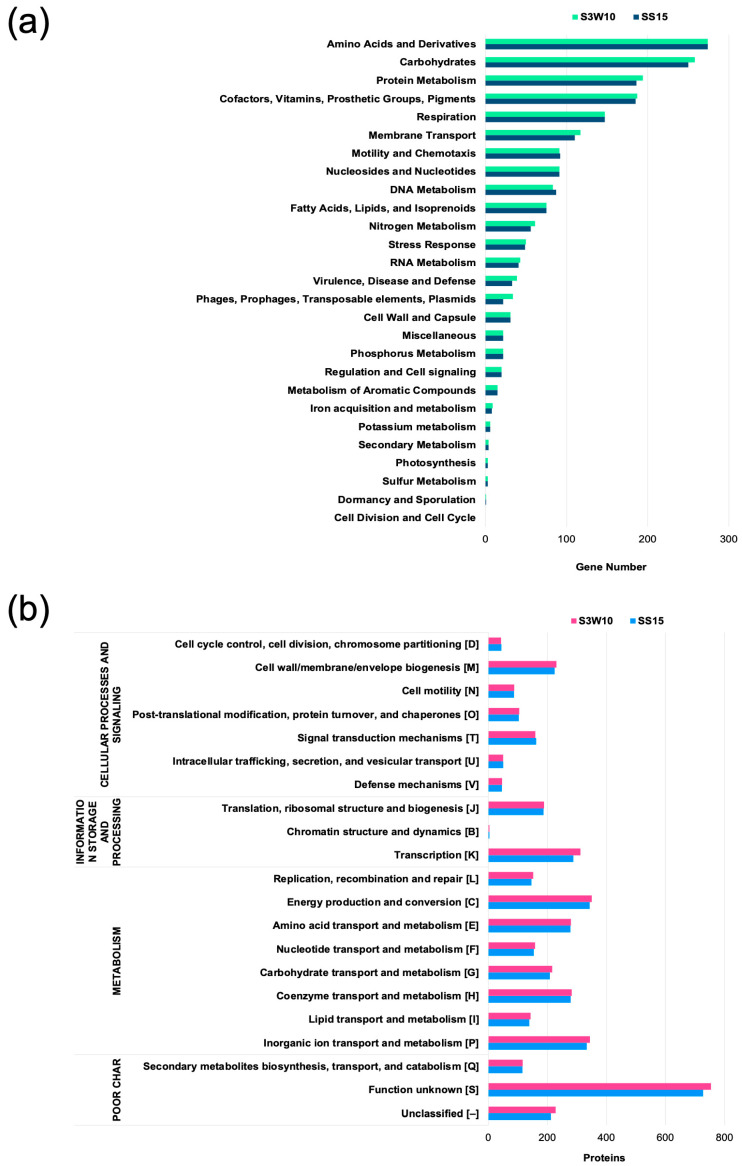
Overview of the distribution of biological subsystems annotated by RAST (**a**) and the functional categorization of proteins based on Cluster of Orthologous Groups (COG) (**b**) in *C. sphaeroides* strains S3W10 and SS15.

The assembled genome of *C. sphaeroides* strain S3W10 is 4.6 Mb, consisting of 130 contigs with a GC content of 69.2%. Strain SS15 has an assembled genome size of 4.4 Mb, with 74 contigs and a GC content of 69.2%, as shown in Figure 1. General genomic information of the draft genomes is provided in Table 1. For functional annotation using RAST subsystems, strain S3W10 was found to have 4260 coding sequences (CDS) assigned to 338 subsystems in the genome, while strain SS15 had 4086 CDS assigned to 336 subsystems. Of these, 1266 (28%) of all CDS for S3W10 and 1151 (27%) for SS15 were classified as hypothetical or of unknown function. Subsystem feature counts of the genomes are presented in Figure 2a. Most subsystems assigned to strains S3W10 and SS15, including amino acids and derivatives (274 for both strains), carbohydrates (258 for S3W10, 250 for SS15), protein metabolism (194 for S3W10, 186 for SS15), and cofactors, vitamins, prosthetic groups, and pigments (187 for S3W10, 185 for SS15). The predicted protein-coded sequences were assigned to 20 COG categories (Figure 2b, Supplementary Table S2). The distribution of proteins assigned to COGs in both genomes showed a similar pattern, indicating a high level of genomic similarity between the two strains.

### 3.2. Mobile Genetic Element Analysis

Research has shown that pathogenic strains possess a higher number of MGEs compared to their probiotic counterparts, suggesting that the genetic mobility of these elements can differentiate pathogenic bacteria from probiotics [33]. One significant aspect of MGEs in probiotics is their association with antibiotic-resistance genes (ARGs). Therefore, careful management is required to prevent the spread of ARG genes to pathogens, as horizontal gene transfer could have unintended consequences for microbial communities.

MobileOG-db was performed to identify MGEs in the S3W10 and SS15 genomes. A total of 161 MGEs were categorized in the S3W10 genome as follows: integration/excision (31), replication/recombination/repair (38), phage (52), stability/transfer/defense (17), and transfer (23). In the SS15 genome, 140 MGEs were identified and categorized into integration/excision (30), replication/recombination/repair (38), phage (33), stability/transfer/defense (19), and transfer (20), as shown in Figure 1.

Bacteriophages, or phages, are viruses that specifically infect bacteria and have recently attracted interest for their role in spreading antibiotic resistance among pathogenic bacteria. While phages can benefit their bacterial hosts by transferring advantageous genes between strains, they may also introduce virulence factors into the bacterial genome [34]. In the genome of strain S3W10, three prophage regions were identified (Figure 1a). All three prophages belong to the taxa Siphoviridae, Myoviridae, and an unknown taxon with region sizes of 4.4 kb, 12.7 kb, and 10.2 kb, respectively. For the SS15 genome, two prophage regions were detected, as shown in Figure 1b. Both prophages are classified as Siphoviridae and Myoviridae with sizes of 9.2 kb and 12.3 kb, respectively. These two families are among the most prevalent bacteriophages found in various aquatic environments including shrimp farms and marine ecosystems [35,36]. The presence of these prophage regions may suggest the potential for horizontal gene transfer which could contribute to the genetic diversity of the bacterial populations [37]. Furthermore, intact prophage regions predicted by the PHASTER server are presented in Supplementary Table S3.

CRISPR and CRISPR/Cas systems function as adaptive immune defenses in bacteria and archaea, providing protection against bacteriophages, plasmids, and other MGEs [38,39]. Analysis of the genomes of *C. sphaeroides* S3W10 and SS15 using the CRISPRCasFinder and CRISPRCasTyper servers revealed a single CRISPR/Cas array with functional activity (Supplementary Figure S1). This array was identified in the genome of *C. sphaeroides* SS15 and classified as a CRISPR-Cas type I-C system. Predicted CRISPR-associated genes (*cas*) included *cas2*_TypeI-II-III, *cas1*_TypeIC, *cas4*_TypeI-II, *cas7c*_TypeIC, *cas8c*_TypeIC, *cas5c*_TypeIC, and *cas3*_TypeI, as depicted in Figure 1.

### 3.3. Identification of Virulence Factors, Antibiotic Resistance Genes and Plasmids

The genome of S3W10 and SS15 strains did not appear to contain any plasmids or virulence factors. In both strains, the VFDB analysis predicted virulence genes related to functions such as adherence, immune evasion, iron uptake, cell surface components, and stress resistance. Genes classified as virulence factors in pathogens often help them tolerate physiological stresses. These genes may enhance the ability of probiotic strains to survive in the gut.

No ARGs were found in the ResFinder 4.1 database (using a 90% threshold and a 60% minimum length) in both genomes S3W10 and SS15. However, the search for AMR genes in the genomes against the CARD database yielded no perfect hits and three and two strict hits for ARGs in S3W10 and SS15, respectively (Supplementary Table S4). The genomes of S3W10 and SS15 were found to carry a resistance gene cluster associated with beta-lactam antibiotic resistance (*ampC* beta-lactamase). This finding aligns with the genome annotations (COG1680) and predictions from the CARD database. The CARD prediction of strain S3W10 was also found to be related to resistance against vancomycin (*vanH*, glycopeptide resistance gene cluster). Nonetheless, the level of expression of these genes remains unknown. Despite the detection of some resistance-related genes, the absence of major ARGs in these probiotic strains suggests a minimal risk of contributing to resistance in clinical settings, supporting their potential safety for use in therapeutic applications

A thorough analysis using Islandviewer 4 did not detect virulence factors or pathogen-associated genes in the genomes of S3W10 and SS15 (Supplementary Table S5). The analysis revealed that the identified genes were distributed across 19 different genomic islands (GIs) in both *C. sphaeroides* genomes, ranging in length from 4270 to 59,441 bp for S3W10 and from 4225 to 41,154 bp for SS15. Most of the CDSs in both genomes were annotated as hypothetical proteins, which are sequences predicted to encode proteins with unknown functions. A significant number of CDSs were identified as stress-related proteins, insertion sequences, antioxidant genes, and transporters. These gene functions support the increased adaptability of the strains in diverse and potentially challenging environments. Moreover, no pathogen-associated genes, virulence factors, or antibiotic-resistance genes were found in the GIs. These results suggest that the strains S3W10 and SS15 are safe for use in aquaculture.

### 3.4. Probiotic Properties of C. sphaeroides Strains

From the previous study, the PNSB strains S3W10 and SS15 offer multiple probiotic benefits for white shrimp (*Litopenaeus vannamei*) cultivation [3,40,41]. These bacteria harbor a diverse array of genes that support the production of proteins crucial for stress response, enabling adaptation to GI conditions such as fluctuations in temperature, pH, bile salts, osmotic pressure, and oxidative stress. Their presence enhances digestion and nutrient absorption while promoting the growth of beneficial gut microbes, contributing to improved digestive health. Furthermore, they produce bioactive compounds such as vitamins, antioxidants, and antimicrobial agents that strengthen the immune system and suppress the pathogen growth of shrimp including *Vibrio* species. In this study, the genomes of S3W10 and SS15 were examined to identify genes linked to these probiotic traits. These genes include those involved in cell adhesion, stress tolerance, vitamin biosynthesis, organic matter degradation, secondary metabolite production, exopolysaccharide (EPS) synthesis, detoxification processes, and nutrient recycling (Table 2 and Table S2).

The probiotic properties of PNSB strains S3W10 and SS15, as demonstrated in previous in vitro and in vivo studies [3,40,41], underscore their potential applications in aquaculture to enhance shrimp growth and health. Strain SS15 is notable for its high protein content (53.98% dry weight) and robust production of digestive enzymes, such as amylase and gelatinase, which improve nutrient digestion and absorption. Although slightly lower in protein content (47.86%), strain S3W10 also produces gelatinase and lipase. Consistent with the genomic analysis of these genomes, we found trehalose synthase/amylase (encoded by *treS* genes) is a bifunctional enzyme with both trehalose synthase and amylase activities [42]. This enzyme converts maltose to trehalose and exhibits amylase-like activity, enabling it to hydrolyze starch. Additionally, the GDSL-like lipase/acylhydrolase family (*tesA*) consists of enzymes crucial for lipid metabolism and the hydrolysis of ester bonds in various substrates [43], while *pepN* (encoding aminopeptidase N) and *degP* (encoding the peptidase S1C family) release free amino acids from proteins [40], thereby enhancing nitrogen availability [44], Both SS15 and S3W10 also produce substantial levels of vitamin B12, which is essential for shrimp health. Vitamin B12 (cobalamin) biosynthesis in these probiotic strains is driven by the *cob*/*cbi* operon genes [45], which encode enzymes involved in the synthesis of this vital nutrient. The production of vitamin B12 by these probiotic *C. sphaeroides* strains enriches the nutritional profile of aquaculture feeds, ensuring that shrimp receive adequate amounts of this key nutrient for optimal growth and health.

Both strains effectively survive and proliferate under simulated shrimp gastrointestinal conditions, demonstrating resilience to pancreatic enzymes [3]. Key adhesion genes, such as *flgK*, *flgL*, *fliC*, and *flgG*, facilitate the establishment of beneficial gut microbiota [46], enabling the bacteria to adhere to the intestinal lining and colonize the host. This adhesion is crucial for probiotic bacteria to effectively modulate the gut ecosystem and outcompete pathogenic microbes [47]. Stress resistance in the S3W10 and SS15 strains is supported by genes that enable them to thrive under adverse environmental conditions. These include genes such as *atpA*, *atpD*, *atpG*, and *atpH*, which facilitate the utilization of proton-translocating ATPase (F0F1-ATPase) for maintaining cytoplasmic pH homeostasis [48]. Additionally, genes like *dnaK*, *dnaJ* (chaperone proteins), and *hslV* (involved in proteolytic processes) help the bacteria maintain proper protein folding and function during periods of heat stress [49].

Genes such as *kdpA*, *kdpB*, and *kdpC* (potassium transport proteins) and *betA*/*betB* (osmoprotectant glycine betaine) assist the bacteria in managing osmotic stress [50], while *otsA* and *otsB* facilitate trehalose biosynthesis [51], aiding in the osmoprotection of the cells. The *kdp* operon has been shown to be induced by osmotic stress in various bacteria, including *Escherichia coli* [52]. In response to osmotic pressure, PNSB may rely on similar mechanisms to regulate potassium levels during osmotic challenges [53]. Furthermore, genes encoding detoxifying enzymes, including *SodB*, *SodC*, and *katE* [54], help neutralize harmful reactive oxygen species, thereby reducing environmental toxins.

Genes involved in nitrogen cycling, including *nifH*, *nifD*, and *nifK*, encode the nitrogenase complex responsible for converting atmospheric nitrogen (N_2_) into ammonia (NH_3_), a process essential for nitrogen fixation [55]. For instance, the transcription of *nifH* is upregulated under anaerobic conditions and in the absence of ammonium, indicating a sophisticated regulatory mechanism that allows *Rhodobacter* to adapt to varying nitrogen availability. The urease enzyme complex, encoded by the *ureA*, *ureB*, and *ureC* genes, facilitates the breakdown of urea into ammonia and carbon dioxide. This process is crucial for nitrogen metabolism, particularly in environments where urea is a significant nitrogen source [56]. The *amtB* gene encodes an ammonium transporter that facilitates the uptake of ammonium ions from the environment [57]. The *norB* gene facilitates the reduction of nitric oxide to nitrous oxide, contributing to denitrification, and *nirK* is responsible for reducing nitrite to nitric oxide [58]. These genes are crucial for managing reactive nitrogen species, which can be toxic to the cells if not adequately detoxified. Overall, these genetic functions contribute significantly to the probiotic benefits of the *C. sphaeroides* strains S3W10 and SS15. These strains not only improve shrimp health and growth but also enhance sustainability in aquaculture practices, underscoring their promising potential as probiotics within the industry.

### 3.5. Carbohydrate-Active enZyme (CAZyme)

Strains S3W10 and SS15 contained 116 and 115 genes encoding CAZymes, respectively (Supplementary Table S6). Both strains exhibited a similar number and distribution of CAZyme families. Glycoside hydrolases (GHs) were the most abundant, with 78 genes in each strain, followed by glycosyltransferases (GTs) with 23 genes and carbohydrate esterases (CEs) with four genes. Additionally, genes related to auxiliary activities (AAs) were found, numbering 10 in S3W10 and 11 in SS15.

A total of 31 GH family proteins were identified using dbCAN in the genomes of strains S3W10 and SS15. The GH13 family was the most prevalent, with nine enzymes identified in both genomes. Genes encoding key enzymes such as β-glucosidase (GH1; EC 3.2.1.21), β-1,4-glucanase (cellulase) (GH8; EC 3.2.1.4), and α-amylase (GH13_3; EC 3.2.1.1) were present in both strains. Enzymes from the GH1 family, particularly β-glucosidases and β-galactosidases, are well-known for their roles in carbohydrate metabolism [59]. β-1,4-Glucanases from GH8 are essential for breaking down β-1,4-glycosidic bonds in polysaccharides, including cellulose and mixed-linkage glucans [60]. GH13 primarily includes enzymes targeting substrates with α-glucoside linkages, such as α-amylase. These linkages are chemical bonds between glucose units in polysaccharides and oligosaccharides. An example of such an enzyme is α-amylase, which hydrolyzes α-1,4-glucosidic bonds found in starch and glycogen, converting them into smaller sugars such as maltose and glucose [61,62]. These enzymes play a crucial role in supporting the metabolic functions of the strains within the shrimp GIT, aiding in the breakdown of sugars such as lactose, sucrose, cellulose, and oligosaccharides [63,64,65].

Previous research has shown that strain SS15 exhibits strong inhibitory effects against pathogenic *Vibrio* species, particularly those causing acute hepatopancreatic necrosis disease (AHPND) [3]. This effect may be attributed to the ability of probiotic bacterial populations to release various substances with bactericidal or bacteriostatic effects on both Gram-negative and Gram-positive bacteria. Proteinaceous substances, such as lysozymes and different types of proteases, act as inhibitory agents that can influence inter-population relationships by affecting competition for resources like chemicals or energy [66,67]. Among these substances, we found lysozyme (GH25; EC:3.2.1.17) and peptidoglycan lytic transglycosylase (GH103; EC 4.2.2.29) in both genomes S3W10 and SS15, which directly degrade the bacterial cell walls of *Vibrio*, weakening the pathogen and preventing infections [68]. These inhibitory substances play a critical role in limiting pathogen proliferation and thereby reduce the pathogen load. Overall, these enzymes work synergistically to enhance shrimp growth and health while creating an unfavorable environment for *Vibrio*, thereby reducing the risk of disease in aquaculture.

### 3.6. Secondary Metabolites Analysis

**Figure 3:**
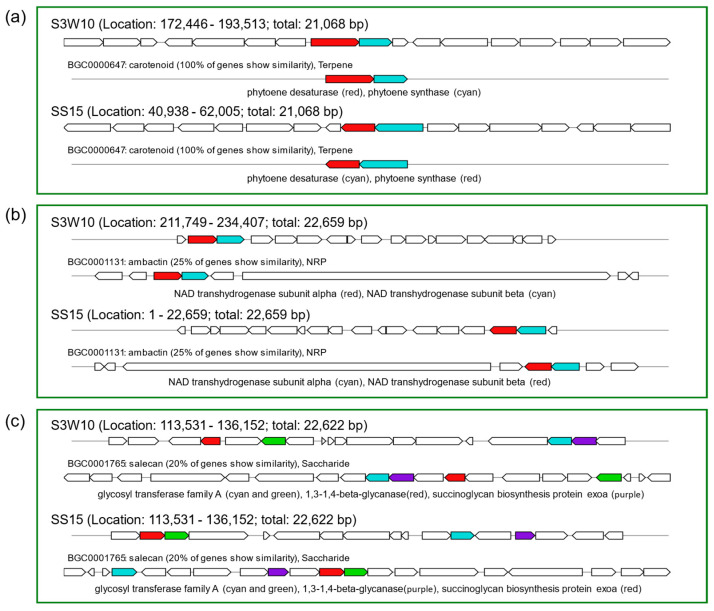
KnownClusterBlast implemented in antiSMASH identified BCGs encoding for (**a**) carotenoid (BGC0000647), (**b**) ambactin (BGC0001131), and (**c**) salecan (BGC0001765) within the genome of *C. sphaeroides* strains S3W10 and SS15.

Secondary metabolite BGCs in the genomes of S3W10 and SS15 were identified using the web-based tools antiSMASH 6.0 and BAGEL4 (Supplementary Table S7). Among the putative gene cluster regions found in both genomes, genes encoding redox cofactors, terpenes, hserlactones, RRE-containing lasso peptides, and type III polyketide synthetases (T3PKS) were identified. Interestingly, the S3W10 and SS15 genomes possess three putative BGCs that matched known BGCs in the MIBiG database at varying percentages of sequence similarity. The identified BGCs were found to be involved in the production of carotenoids (100% similarity), ambactin (25% similarity), and salecan (20% similarity), as illustrated in Figure 3. However, analysis using the BAGEL4 web server did not reveal any genetic markers for bacteriocins or ribosomally synthesized and post-translationally modified peptides (RiPPs) in the genomes of strains S3W10 and SS15.

These matched BGCs were found to be involved in the biosynthesis of carotenoids (100% similarity), ambactin (25% similarity), and salecan (20% similarity), as illustrated in Figure 3. However, analysis using the BAGEL4 web server did not reveal any genetic markers for bacteriocins or RiPPs in the genomes of strains S3W10 and SS15.

PNSB are known for their ability to produce carotenoids, which are essential for the coloration and health of crustaceans, as they cannot synthesize these compounds endogenously [69]. The incorporation of carotenoids from PNSB into shrimp diets not only improves pigmentation but also enhances immune responses and stress tolerance, which are critical factors in aquaculture management [70]. Research has shown that dietary supplementation with carotenoids can lead to significant improvements in shrimp growth performance. For instance, a study indicated that shrimp fed with carotenoid-enriched diets exhibited increased biomass production and enhanced coloration, which is a desirable trait in the aquaculture market [71]. The carotenoid biosynthetic pathway in *C. sphaeroides* includes several key genes, such as *crtE*, *crtB*, *crtC*, *crtD*, *crtI*, and *crtF*, which are responsible for the conversion of geranylgeranyl pyrophosphate into carotenoids like lycopene and spheroidene [72].

Additionally, a known nonribosomal peptide (NRP) cluster was identified as being involved in the biosynthesis of ambactin, a hexapeptide recognized as a bioactive compound derived from *Xenorhabdus miraniensis* DSM 17902, exhibiting activity against protozoa [73]. Furthermore, salecan, a soluble β-glucan from *Agrobacterium* sp. ZX09, demonstrates various beneficial properties, including immunomodulatory effects and potential applications as a food additive or in drug delivery systems [74,75]. Research indicates that salecan can positively influence the intestinal health of shrimp by modulating the gut microbiota and inhibiting inflammatory responses. A study demonstrated that dietary supplementation with β-glucan, including salecan, led to improved immune function and antioxidant capacity in shrimp, suggesting its role as an effective immunostimulant [76].

### 3.7. Pan-Genome of C. sphaeroides

**Figure 4:**
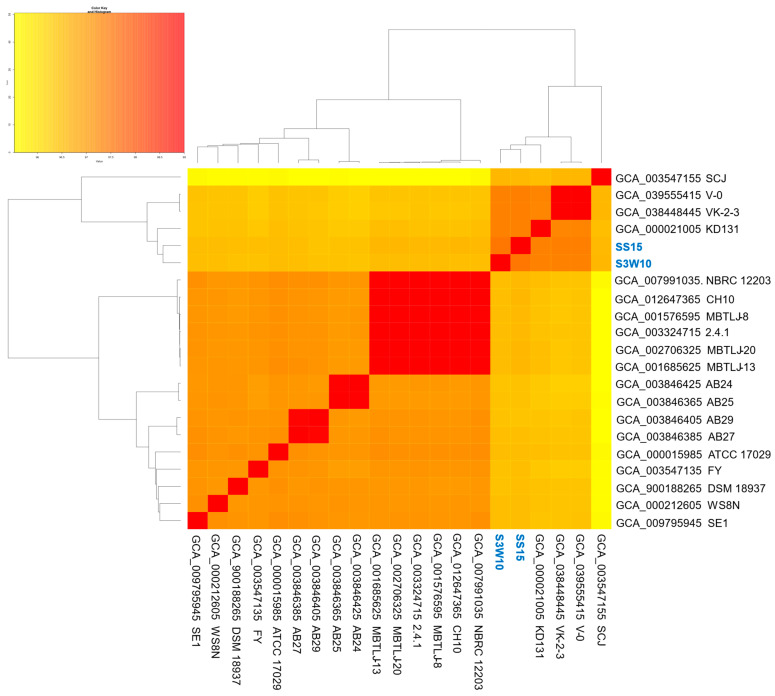
Heatmap illustrating genomic relatedness based on ANI values among the *C. sphaeroides* genomes analyzed in this study. The color scale represents the range of genomic similarity, with strains S3W10 and SS15 prominently highlighted.

From the 35 genomes currently classified as *C. sphaeroides* (NCBI dataset, 20 October 2024), 20 were selected after excluding unannotated, redundant, or atypical genomes. These selected genomes underwent quality control checks for sequencing, assembly, and taxonomy before being analyzed using PanACoTA software. While the exclusion criteria reduced the number of genomes analyzed, they ensured greater accuracy, reliability, and consistency in the study. Using an ANI cutoff of 95–96% for species-level classification [77], 19 of the 20 genomes were confirmed as *C. sphaeroides*, showing ANI values above 96% (Supplementary Table S1). ANI analysis further revealed that the genomes of *C. sphaeroides* S3W10 and SS15 clustered closely with the VK-2-3, V-0, and KD131 strains (Figure 4).

**Figure 5:**
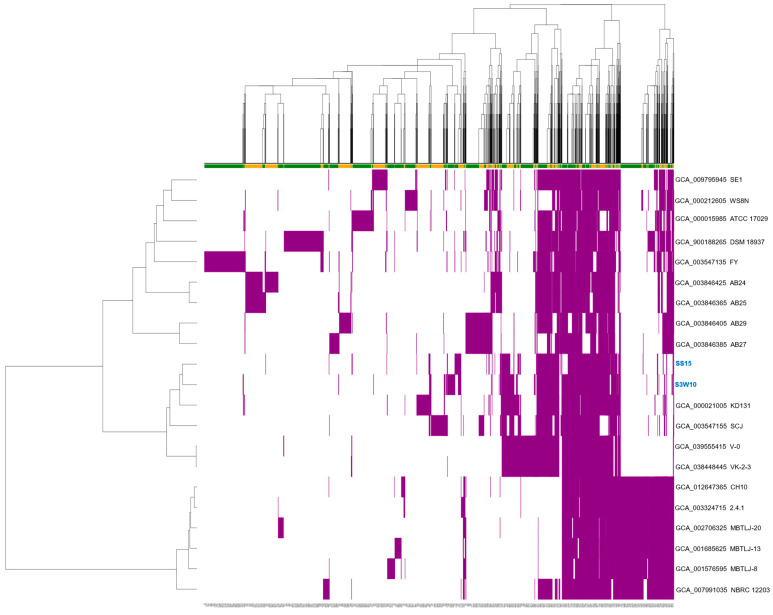
Overview of presence/absence variation (PAV) of *C. sphaeroides* genomes (n = 21). Core genes are not included. Strains S3W10 and SS15 are prominently highlighted.

The PanACoTA-based pan-genome analysis of S3W10 and SS15, together with *C. sphaeroides* genomes from NCBI, identified a total of 7918 clusters, including 3096 core genes (39.1%), 2640 dispensable gene clusters (33.3%), and 2182 strain-specific genes (27.6%). These results were based on an 80% sequence identity threshold in BLAST pairwise alignments. The rarefaction curve, which shows the number of protein clusters identified as more genomes are added, showed an α value of 0.73 (Supplementary Figure S2). The α value below 1 indicates an open pan-genome according to Heap’s Law [78]. As more genomes were included, the pan-genome size continued to grow, supporting that this species has an open pan-genome. The core genome accounted for only 39.1% of the total pan-genome, consistent with other open-genome species like *Rhodococcus equi* (32%) [79]. This analysis indicates that *C. sphaeroides* strains have high genomic diversity. Species with a free-living lifestyle and adaptability often exhibit open pan-genomes, which enable them to adapt and thrive in a wide range of ecological environments. Furthermore, the sharing patterns of dispensable gene clusters (Figure 5) were consistent with the results of the ANI-based phylogenetic analysis.

Among the dispensable clusters, the strains CH10, 2.4.1, MBTLJ-20, MBTLJ-13, MBTLJ-8, and NBRC 12203 shared a large number of clusters, accounting for 256 (Supplementary Figure S3). *C. sphaeroides* strains S3W10 and SS15 shared 92 clusters with the strains SE, ATCC 17029, DSM 18937, FY, AB24, AB25, AB29, KD131, SCJ, V-0, VK-2-3, and NBRC 12203, and other 62 genes with all *C. sphaeroides* strains except ATCC 17029 and SCJ (Supplementary Figure S3). The distribution of strain-specific clusters revealed that strains FY, DSM 18937, and ATCC 17029 had the highest number of unique genes with 395, 376, and 188 singletons, respectively. In contrast, the strains VK-2-3, V-0, and 2.4.1 contained the fewest singletons, with only 3, 5, and 19, respectively (Supplementary Figure S4). Most other strains had between 25 and 126 unique clusters. Among these, strains S3W10 and SS15 contained the number of singletons—71 and 32, respectively.

The functional classification of the pan-genome, based on the distribution of COG categories, revealed that many gene clusters were associated with amino acid transport and metabolism (E), energy production and conversion (C), transcription (K), carbohydrate transport and metabolism (G), and inorganic ion transport and metabolism (P) (Supplementary Figure S5). Furthermore, the phylogenetic tree constructed using hierarchical clustering of the binary matrix representing the presence or absence of accessory genes showed that strains S3W10 and SS15 grouped within the same clade as the VK-2-3, V-0, and KD131 strains (Supplementary Figure S6).

### 3.8. Phylogenomic of C. sphaeroides Species

**Figure 6:**
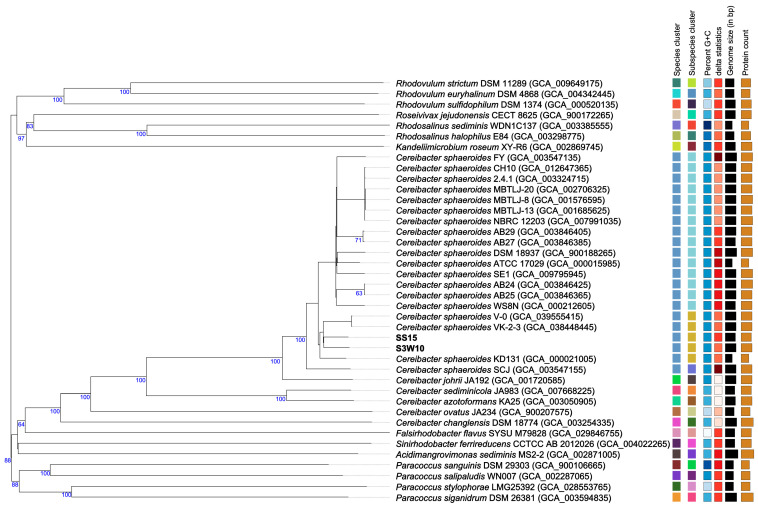
The phylogenomic tree for the *C. sphaeroides* species inferred using FastME 2.1.6.1 based on GBDP distances computed from genome sequences. Branch lengths are scaled according to the GBDP distance formula *d5*. Support values greater than 60% from 100 pseudo-bootstrap replications are indicated below the branches, with an average branch support of 51.3%. Strains S3W10 and SS15 are prominently highlighted.

The phylogenomic analysis was conducted through genome–genome comparisons using the TYGS server. In this analysis, 21 *C. sphaeroides* genomes were submitted to the TYGS, which automatically included 19 related type strains for comparison, as shown in Supplementary Table S8. The pairwise dDDH results were used to construct a phylogenomic tree (Figure 6), which had an average branch support of 51.3% and a δ statistic of 0.159. The strains of *C. sphaeroides* formed a species cluster with dDDH values ≥ 70%, including the type strain *C. sphaeroides* 2.4.1. The genomes were categorized into three subspecies groups of *C. sphaeroides*. Strains S3W10 and SS15 were surrounded by subclusters containing the KD131 strain, as well as the VK-2-3 and V-0 strains. These findings confirm the position of strains S3W10 and SS15 within the species *C. sphaeroides* and clarify the taxonomic classification of this species. Moreover, the results obtained using ANI, dDDH, and dispensable cluster-sharing patterns suggest that this combined approach is a reliable method for defining the taxonomic structure of *C. sphaeroides*.

## 4. Conclusions

Our study provides a comprehensive genomic analysis of two *C. sphaeroides* strains, S3W10 and SS15, isolated from shrimp ponds, highlighting their potential as probiotics in aquaculture. The draft genomes of both strains were characterized, revealing essential genetic features that support their probiotic functions, including stress tolerance, antioxidant activity, and enhanced nutrient absorption capabilities. Notably, the presence of various genes related to the production of digestive enzymes and vitamin B12 aligns with previous in vitro and in vivo findings, supporting their functional benefits in aquaculture. Importantly, the identification of mobile genetic elements, virulence factors, and antimicrobial resistance underscores their safety as probiotics for applications in aquaculture. The comparative genomic analysis revealed an open pan-genome for the species, positioning S3W10 and SS15 phylogenetically within the *C. sphaeroides* species. Overall, these strains exhibit significant probiotic properties that can enhance shrimp farming sustainability by improving health outcomes and potentially reducing the need for antibiotics. This study supports the use of *C. sphaeroides* strains S3W10 and SS15 for probiotic applications in aquaculture.

## Figures and Tables

**Table 1:** Genomic features of strains S3W10 and SS15.

Attribute	S3W10	SS15
Genome size (bp)	4,597,894	4,413,552
Contigs	130	74
DNA G + C content (%)	69.2	69.2
Protein coding sequences (CDSs)	4524	4294
ANIb (%) with SS15	98.29	100
rRNA	3	3
tRNA	52	51
tmRNA	1	1
RAST subsystems	338	336

**Table 2:** List of probiotic marker genes identified in S3W10 and SS15.

Gene	Product/Description	Gene Number	
		S3W10	SS15
**Adhesion**			
*flgK*	Flagellar hook-associated protein FlgK	1	1
*flgL*	Bacterial flagellin C-terminal helical region	1	1
*fliC*	Flagellin subunit protein	2	2
*flgG*	Flagellar basal-body rod protein FlgG	3	3
*lspA*	Lipoprotein signal peptidase	1	1
*msrA*	Peptide methionine sulfoxide reductase	1	1
**Acid stress**			
*atpA*, *atpD*, *atpG*, *atpH*, *atpC*	FoF1-type ATP synthase (alpha, beta, gamma, delta, epsilon) subunit	2, 2, 3, 1, 2	2, 2, 3, 1, 2
*atpE*	FoF1-type ATP synthase subunit K	2	2
*aspS*	Aspartyl-tRNA synthetase	1	1
**Heat stress**			
*dnaK*	DnaK chaperone protein	3	2
*dnaJ*	DnaJ chaperone protein	2	2
*grpE*	Chaperonin GroEL (HSP60 family)	1	1
*groES*	Co-chaperonin GroES (HSP10)	1	1
*ibpB*	Small heat shock protein (HSP20) family	1	1
*hslV*, *hslU*	ATP-dependent protease HslVU (ClpYQ)	1, 1	1, 1
**Osmotic pressure stress**			
*kdpA*, *kdpB*, *kdpC*	K+-transporting ATPase, A, B, C chain	1, 1, 1	1, 1, 1
*betA*, *betB*	Involved in the biosynthesis of the osmoprotectant glycine betaine	1, 2	1, 2
*otsA*, *otsB*	Involved in the osmoprotection via the biosynthesis of trehalose	1, 1	1, 1
**Oxidative stress**			
*sodB*, *sodC*	Destroys radicals that are normally produced within the cells and which are toxic to biological systems	1, 1	1, 1
*katE*	Catalase (COG0753)	1	1
*tlpA*	Alkyl hydroperoxide reductase	1	1
*trxA*	Thioredoxin reductase	1	1
*msrA*	Repair enzyme for proteins that have been inactivated by oxidation	1	1
**Bile resistance**			
*arsB*	Bile acid sodium symporter	1	1
**Photosynthesis**			
*pufL*	Photosynthetic reaction center L subunit	1	1
*pufA*, *pufB*	Antenna complexes are light-harvesting systems, which transfer the excitation energy to the reaction centers	1, 1	1, 1
*rbcL*	Ribulose 1,5-bisphosphate carboxylase large subunit	1	1
**Lactate synthesis**			
*ldhA*	Lactate dehydrogenase or related 2-hydroxyacid dehydrogenase	1	1
**Vitamin biosynthesis**			
*cobS*, *cobN*, *cobU*, *cobT*	*cob*/*cbi* operon for cobalamin (vitamin B12) biosynthesis	1, 1, 1, 1	1, 1, 1, 1
*pdxA*, *pdxJ*, *pdxH*, *pdxK*	pdx operon for vitamin B6 biosynthesis	2, 1, 1, 1	2, 1, 1, 1
**Organic matter degradation**			
*treS*	Trehalose synthase/amylase TreS	1	1
*tesA*	GDSL-like Lipase/Acylhydrolase family	1	1
*degP*	Peptidase S1C family with trypsin-like peptidase domain (COG0265)	3	3
*pepN*	Aminopeptidase N	1	1
*dcp*	PFAM peptidase M3A and M3B, thimet oligopeptidase F	1	1
*pepF*	TIGRFAM oligoendopeptidase, pepF M3 family	1	1
**Carotenoid biosynthesis**			
*crtF*	Demethylspheroidene O-methyltransferase	1	1
*crtE*	Encodes geranylgeranyl diphosphate (GGPP) synthase, which synthesizes GGPP, a precursor for carotenoid biosynthesis	1	1
*crtC*	Involved in the biosynthesis of carotenoids spheroidene and spirilloxanthin	1	1
*crtB*	Encodes phytoene synthase, which converts GGPP into phytoene	1	1
*crtI*	Converts phytoene into all-trans-neurosporene as the major product, through intermediates phytofluene and zeta-carotene	1	2
*crtA*	Spheroidene monooxygenase, involved in the hydroxylation of spheroidene, contributing to carotenoid diversity	1	1
*tspO*	Part of the TspO MBR family, involved in carotenoid biosynthesis	1	1
**Exopolysaccharide (EPS) biosynthesis**			
*wza*	Polysaccharide biosynthesis/export protein	1	1
*galU*	UTP-glucose-1-phosphate uridylyltransferase	1	1
*galE*, *manC*	Involved in sugar precursor synthesis for EPS	4, 1	4, 1
**Nitrogen removal and recycling**			
*amtB*	Ammonia channel protein AmtB	1	1
*gdhA*	Glu, Leu, Phe, Val dehydrogenases family	1	1
*glnA*	Glutamine synthetase	1	1
*norB*	Nitric oxide reductase large subunit	1	1
*nirK*	Nitrite reductase	1	1
*cysI*	Nitrite and sulfite reductase 4Fe-4S	1	1
*ureA*, *ureB*, *ureC*	Urease gamma, beta, alpha subunit	1, 1, 1	1, 1, 1
*ureE*, *ureF*	Urease accessory protein (UreE, UreF)	1, 1	1, 1
*nifH*	Nitrogenase subunit NifH	2	1
*nifD*	Nitrogenase molybdenum-iron protein, alpha chains	2	2
-	COG3381: Nitrate reductase delta subunit	1	1
**Phosphorus removal and recycling**			
*ppx*	Inorganic pyrophosphatase/exopolyphosphatase	1	1
*ppk2*	Polyphosphate kinase 2, PPK2 family	1	1
*pstB*	ABC-type phosphate transport system, ATPase component	1	1
*pstA*, *pstC*	ABC-type phosphate transport system, permease component	1, 1	1, 1
*pstS*	ABC-type phosphate transport system, periplasmic component	1	1
**Hydrogen sulfide detoxification**			
*cysK*	Cysteine synthase	1	1
*cysE*	Serine acetyltransferase	1	1
*cysI*	Sulfite reductase, beta subunit (hemoprotein)	1	1

## Data Availability

The GenBank accession numbers for the genomes of strains SS15 and S3W10 are JBAJND000000000 and JBAJNC000000000, respectively.

## Supplementary Materials

The following supporting information can be downloaded at: https://www.mdpi.com/article/10.3390/life14121691/s1, Figure S1: CRISPR/Cas system of *C. sphaeroides* SS15 as determined by using the CRISPRCasTyper online server.; Figure S2: Presents the rarefaction curve, which shows the cumulative number of gene clusters computed by micropan R package.; Figure S3: Upset diagram for accessory genes in *C. sphaeroides* strains.; Figure S4: Distribution of strain-specific genes in the pan-genome analysis of *C. sphaeroides* strains.; Figure S5: Pan-genome analysis of *C. sphaeroides* strains.; Figure S6: Pan-genome phylogenetic tree of *C. sphaeroides* strains.; Table S1: The summary of the *Cereibacter sphaeroides* genomes from NCBI database.; Table S2: Genome annotation of *Cereibacter sphaeroides* strains S3W10 and SS15.; Table S3: Summary of prophage regions identified in the S3W10 and SS15 genomes.; Table S4: List of CARD resistance gene identifier found in the genomes S3W10 and SS15.; Table S5: Genomic islands found in the *Cereibacter Sphaeroides* genomes S3W10 and SS15.; Table S6: CAZy annotation of *C. sphaeroides* genomes S3W10 and SS15.; Table S7: Predicted biosynthetic gene clusters of the *Cereibacter sphaeroides* strains S3W10 and SS15.; Table S8: The 19 type strains automatically included as the closest neighbors to the *C. sphaeroides* genomes in TYGS sever.
